# Clinical and laboratory profiles of patients with early spontaneous healing in cutaneous localized leishmaniasis: a historical cohort study

**DOI:** 10.1186/s12879-017-2658-4

**Published:** 2017-08-10

**Authors:** Carla Oliveira-Ribeiro, Maria Inês Fernandes Pimentel, Raquel de Vasconcellos Carvalhaes Oliveira, Aline Fagundes, Maria de Fatima Madeira, Cintia Xavier Mello, Eliame Mouta-Confort, Claudia Maria Valete-Rosalino, Erica de Camargo Ferreira Vasconcellos, Marcelo Rosandiski Lyra, Leonardo Pereira Quintella, Liliane de Fatima Antonio, Armando Schubach, Fatima Conceição-Silva

**Affiliations:** 1 Laboratório de Imunoparasitologia, Instituto Oswaldo Cruz IOC/FIOCRUZ, Pavilhão 26 – 4° andar, sala 406-C, Av. Brasil 4365, Manguinhos, Rio de Janeiro, RJ 21040-360 Brazil; 2 Laboratório de Pesquisa Clínica e Vigilância em Leishmanioses - LAPCLIN VIGILEISH, Instituto Nacional de Infectologia Evandro Chagas INI/FIOCRUZ, Rio de Janeiro, Brazil; 3 Laboratório de Epidemiologia Clínica, Instituto Nacional de Infectologia Evandro Chagas INI/FIOCRUZ, Rio de Janeiro, Brazil; 4 Jovem Cientista do Nosso Estado, Fundação Carlos Chagas Filho de Amparo à Pesquisa no Estado do Rio de Janeiro (FAPERJ), Rio de Janeiro, Brazil; 5 Departamento de Otorrinolaringologia e Oftalmologia, Faculdade de Medicina, Universidade Federal do Rio de Janeiro, Rio de Janeiro, Brazil; 6 Serviço de Anatomia Patológica- SEAP, Instituto Nacional de Infectologia Evandro Chagas. INI/FIOCRUZ, Rio de Janeiro, Brazil; 7 Fellow Researcher of the Conselho Nacional de Desenvolvimento Científico e Tecnológico (CNPq), Rio de Janeiro, Brazil; 8 Cientista do Nosso Estado, Fundação Carlos Chagas Filho de Amparo à Pesquisa no Estado do Rio de Janeiro (FAPERJ), Rio de Janeiro, Brazil

**Keywords:** American tegumentary leishmaniasis, *Leishmania (Viannia) braziliensis*, Clinical cure, Spontaneous healing, Diagnosis

## Abstract

**Background:**

Skin ulcers in American cutaneous leishmaniasis (ACL) may heal spontaneously after months/years. However, few cases may present quick heal even during diagnosis procedure (early spontaneous healing- ESH). The main objective of this study was to compare ESH patients with cases requiring specific treatment [non-ESH (NESH)].

**Methods:**

A historical cohort study of ACL patients (*n* = 445) were divided into 2 groups: ESH – spontaneously healed patients (*n* = 13; 2.90%), and NESH- treated patients (*n* = 432; 97.10%). We compared clinical and laboratorial findings at diagnosis, including the lesion healing process.

**Results:**

ESH patients had a higher percentage of single lesions (*p* = 0.027), epithelialized lesion on initial examination (*p* = 0.001), lesions located in the dorsal trunk (*p* = 0.017), besides earlier healing (*p* < 0.001). NESH presents higher frequency of ulcerated lesions (*p* = 0.002), amastigotes identified in histopathology exams (*p* = 0.005), positive cultures (*p* = 0.001), and higher positivity in ≥3 parasitological exams (*p* = 0.030). All ESH cases were positive in only a single exam, especially in PCR.

**Conclusions:**

ESH group apparently presented a lower parasitic load evidenced by the difficulty of parasitological confirmation and its positivity only by PCR method. The absence or deficiency of specific treatment is commonly identified as predisposing factors for recurrence and metastasis in ACL. However, due to the drugs toxicity, the treatment of cases which progress to early spontaneous healing is controversial. ESH patients were followed for up to 5 years after cure, with no evidence of recrudescence, therefore suggesting that not treating these patients is justifiable, but periodic dermatological and otorhinolaryngological examinations are advisable to detect a possible relapse.

## Background

Leishmaniasis comprises a group of infectious diseases that may occur with cutaneous or visceral involvement. They are caused by protozoa of the genus *Leishmania* transmitted to humans by infected female sandflies. American cutaneous leishmaniasis (ACL), cutaneous presentantions in the New World, clinically presents quite variable lesions, from the acneiform type to ulcers with or without lymphadenopathy. The typical cutaneous lesion is an ulcer (usually a single one) with infiltrated borders in exposed areas of the body, but clinical presentation may vary depending on the immune status of the host, the parasite load, and the involved *Leishmania* species [1]. Treatment is indicated in confirmed cases but the recommended drugs are quite toxic [2–4], and in recent years cases of resistance have been described [5–8].

The typical ulcer of cutaneous leishmaniasis (CL) can progress to cure over a period of several months to years, if not diagnosed and treated [9]. However, some patients have early spontaneous resolution of the disease; they are diagnosed according to clinical and laboratory criteria and, before the start of drug therapy, the lesions begin the process of clinical cure, and may not require treatment [10, 11].

The Leishmaniasis Surveillance Laboratory (LAPCLIN VIGILEISH) at Evandro Chagas National Institute of Infectious Diseases (INI), Oswaldo Cruz Foundation (Fiocruz), Rio de Janeiro, receives patients with suspected ACL from the state of Rio de Janeiro and from other regions of Brazil. Patients with early clinical signs of healing without specific treatment and those treated are invited to participate in a follow-up of at least 5 years with dermatological and otorhinolaryngological annual exams, creating conditions for prompt identification of signs of recurrence of skin lesions and/or development of mucosal involvement. This study allowed the comparison of clinical, laboratory and follow-up data of the leishmaniasis patients with early spontaneous healing with those of the patients that needed specific treatment.

## Methods

### Group formation and ethical considerations

This is a historical cohort study of 445 patients with cutaneous leishmaniasis attended at LAPCLIN VIGILEISH from November, 2002 to December, 2013. This study was approved by the Ethics Committee of INI/Fiocruz under number 19700413.6.0000.5262. All participants signed an informed consent form. Patients under 18 had the informed consent form signed by parents or guardians who accompanied them during all procedures.

Diagnosis of ACL through clinical (medical history, morphology and topography of skin lesion, and compatible evolution time prior to the diagnosis), epidemiological (origin and age) data and/or parasitological confirmation were considered as inclusion criteria.

**Fig. 1:**
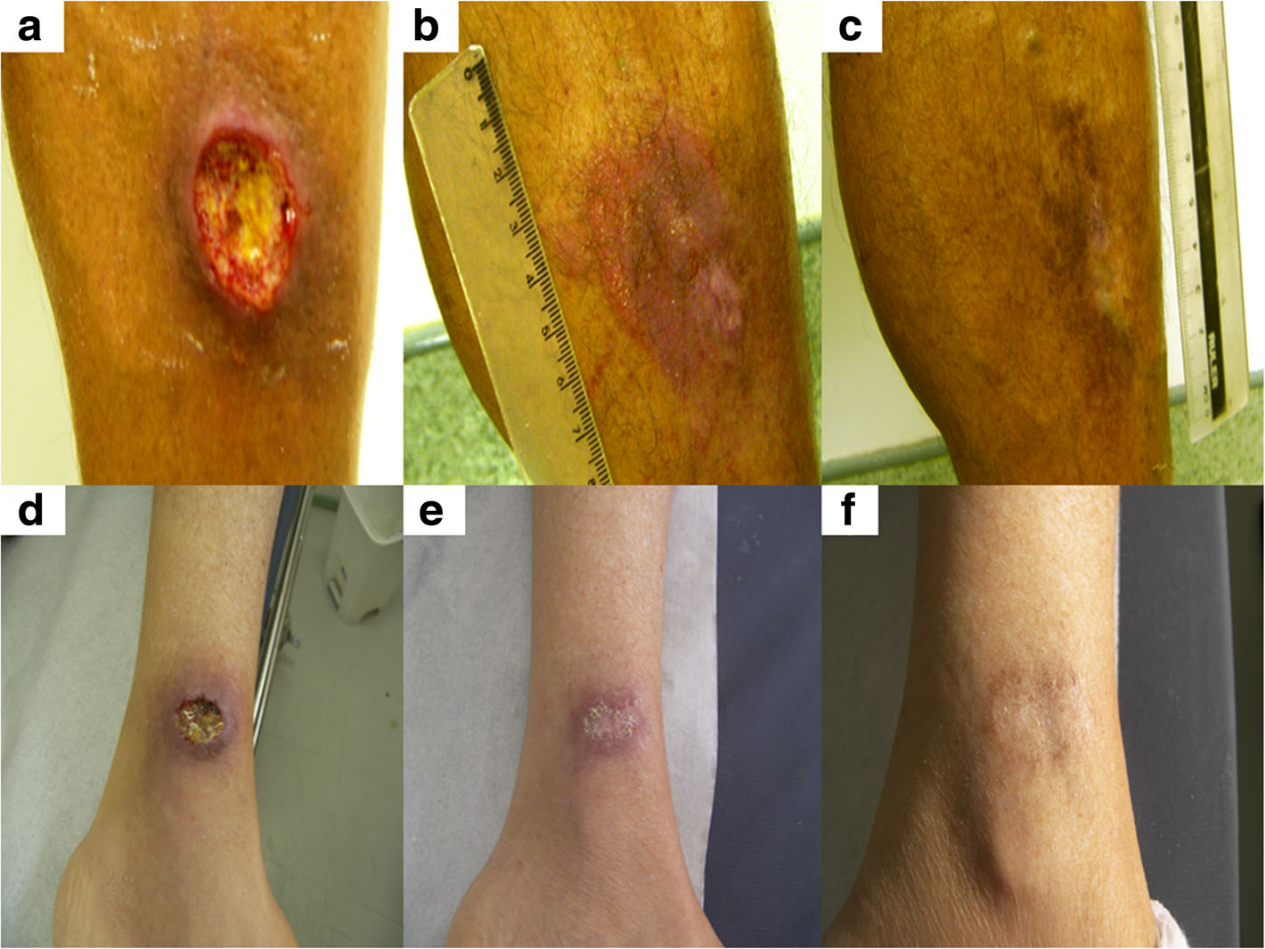
American cutaneous leishmaniasis (ACL), typical skin lesion, non-early spontaneous healing(NESH). **a** Pretreatment; **b** Epithelialization; **c** Wound healing. ACL, early spontaneous healing (ESH). **d** Before diagnosis; **e** Epithelialization; **f** Healing

Patients were classified into one of two groups: ESH - patients with clinical and laboratory diagnosis of cutaneous leishmaniasis with early spontaneous healing (*n* = 13); NESH- patients with typical cutaneous leishmaniasis requiring specific treatment (*n* = 432) (Fig. 1).

An ESH case was defined as a patient with a positive parasitological exam for *Leishmania* spp. and epithelialized lesion(s) without crusts in the period between the laboratory investigation for the disease diagnosis and the expected date for the start of the specific treatment [12]. The morphology of included lesions were ulcer/ulceration, papule, nodule or plaque [12]. The lesion should be present in a current context of illness.

We excluded patients with 10 or more lesions, those who did not sign the informed consent form, those who have received prior treatment for ACL or that abandoned treatment, those who were using immunosuppressive drugs, patients with diseases with altered immune profiles (such as HIV infection), pregnant women, and patients with mucosal lesion forms at diagnosis.

### Evaluation of the clinical and laboratory profiles of the patients

All patients underwent clinical, dermatologic, otorhinolaryngological and laboratory evaluation, the last one comprising the following tests to confirm the diagnosis: Montenegro skin test (MST); indirect immunofluorescence assay (IFA) and/or immunoenzymatic assay (ELISA) in blood samples; culture for *Leishmania sp.* isolation, imprint, histopathology, polymerase chain reaction (PCR) [13] and anti-*Leishmania* immunohistochemistry (IHC) in fragments of the cutaneous lesion obtained throught biopsy procedure. MST was evaluated with respect to its largest diameter, as negative (<5 mm) or positive (≥ 5 mm). The samples were obtained at the first appointment. Patients had clinical (dermatological and otorhinolaryngological) and laboratory (serology for *Leishmania* spp.) examinations annually for up to five years.

Analysis considered sociodemographic variables (sex, age and origin); use of non-specific medications (oral/topic antibiotics, hypoglycemics, antihypertensive and hypocholesterolemic drugs); data on past medical history including clinical comorbidities (hypertension, diabetes mellitus, heart disease, dyslipidemia, among others); characteristics of the lesions (number, duration, size, morphology and topography); results of laboratory tests; and the outcomes of spontaneous healing or need for specific treatment.

### Comparative analysis of the clinical and laboratory profiles of the patients

The softwares Statistical Package for the Social Sciences (SPSS Inc., USA) version 16.0, and R version 2.15 (R Foundation for Statistical Computing, Vienna, Austria) were used. Data were descriptively analyzed using frequency for categorical variables; and median, minimum and maximum for continuous variables. The association of qualitative variables according to the groups was assessed using Fisher’s Exact test. The Kolmogorov-Smirnov test indicated rejection of the normality of the continuous variables. The comparison of continuous variables in ESH and NESH groups was performed using the Mann-Whitney test.

We used survival analysis to investigate differences in healing time of the cutaneous lesion between the studied groups, considering the time until the occurrence of the outcome or censoring. The event “clinical cure of lesion” was considered the outcome, and defined as epithelized lesion(s) without crusts. Censoring was applied when the outcome was not observed at the end of the follow-up period. ESH and NESH patients with missing data in the medical records regarding time to heal were excluded from the survival analysis (*n* = 10); therefore, 9 patients from the ESH group and 426 patients from NESH were included in this analysis. We performed the analysis based on the healing time (total time elapsed between the date of the first medical visit and the date of the complete healing of the lesion). The non-parametric method of Kaplan-Meier allowed the analysis of probabilities for survival over time. Peto and/or Log-rank tests were used to check for differences between the stratified survival curves.

*P*-values <0.05 indicated statistically significant tests.

## Results

Four hundred and forty-five patients were analyzed, with a median age of 37 (15–80) years, and 63.40% were men. Most patients (64.50%) came from the state of Rio de Janeiro (Brazil).

Considering all the studied cases, 64.70% of the patients had a single lesion. Lesions affected different sites of the body: exclusive on the lower limbs (35.10%); exclusive on the upper limbs (30.10%); restricted to regions of head and trunk (21.80%); and multiple locations (13.00%). The presence of ulcers was prevalent at diagnosis (*n* = 394; 88.50%), but in 9 cases epithelialized lesions were observed in the initial clinical examination (2.00%). The median of the largest diameter of the lesions was 30 mm (4–120 mm). The median MST measure was 17 mm (2–71 mm), and 82.50% of the patients were considered positive in this exam. The median time of progression of the lesion previous to diagnosis was 60.87 days; and the median follow-up period of all cohort was 803 days. The median time between the first and the second medical appointment was 29 days in the whole studied cohort. The median follow-up period of NESH group was 990 days and in the ESH group was 631 days.

Four hundred and thirty-two patients (97.10%) were treated for ACL, while in thirteen individuals (2.90%) the spontaneous healing phenomenon was observed. In the NESH group, 430 patients were treated with meglumine antimoniate (99.53%), and 2 cases were treated with amphotericin B deoxycholate (0.47%).

The median time to lesion healing was lower in the ESH group (35 days) compared to the NESH group (77 days), *p* = 0.001. Patient characteristics according to the outcomes of spontaneous resolution or need for specific treatment are shown in Table 1.

**Table 1:** Distribution of the clinical and laboratorial characteristics of 445 patients with cutaneous leishmaniasis according to the need for treatment (NESH) or no treament (ESH), INI/Fiocruz, Brazil (2002–2013)

Variable	Status	Treatment/No treatment				
		NESH		ESH		*p*-value*
		n	%	n	%	
*SOCIODEMOGRAPHIC VARIABLES*						
Sex	Male	277	64.1	5	38.5	0.057
	Female	155	35.9	8	61.5	
Age group	15–30 years	150	34.7	5	38.5	0.877
	> 30 years	282	65.3	8	61.5	
Comorbities	Yes	115	26.6	2	15.4	0.291
	No	317	73.4	11	84.6	
Medications	Yes	275	63.7	7	53.8	0.327
	No	157	36.3	6	46.2	
*CHARACTERISTICS OF THE LESIONS*						
Number of lesions	1	276	63.9	12	92.3	**0.027**
	2 or more	156	36.1	1	7.7	
Larger diameter of the lesion (mm)	4--26	167	39.2	6	50.0	0.320
	≥ 27	259	60.8	6	50.0	
Ulcer	Yes	387	89.6	7	53.8	**0.002**
	No	45	10.4	6	46.2	
Epithelialized lesion at first visit	Yes	5	1.2	4	30.8	**0.001**
	No	427	98.8	9^a^	69.2	
Location at the dorsal aspect of the trunk	Yes	33	7.6	4	30.8	**0.017**
	No	399	92.4	9	69.2	
MST with bullous reaction	Yes	10	2.3	2	15.4	**0.044**
	No	422	97.7	11	84.6	
*LABORATORY EXAMS*						
MST	Positive	355	95.7	12	92.3	0.450
	Negative	16	4.3	1	7.7	
IFA	Positive	278	80.4	6	66.7	0.260
	Negative	68	19.6	3	33.3	
ELISA	Positive	341	90.0	9	90.0	0.736
	Negative	38	10.0	1	10.0	
CULTURE	Positive	341	84.6	1	9.1	**0.001**
	Negative	62	15.4	10	90.9	
IMPRINT	Positive	133	42.1	1	11.1	0.142
	Negative	183	57.9	8	88.9	
PCR	Positive	124	86.1	5	62.5	0.102
	Negative	20	13.9	3	37.5	
*HISTOPATHOLOGY*						
AMASTIGOTES	Yes	266	62.6	3	23.1	**0.005**
	No	159	37.4	10	76.9	
*IMMUNOHISTOCHEMISTRY*						
AMASTIGOTES	Yes	12	50.0	3	25.0	**0.038**
	No	12	50.0	9	75.0	

NESH- cutaneous leishmaniasis cured after treatment; ESH - patients with early spontaneous healing of the lesions without specific treatment

*MST* Montenegro skin test; *IFA* indirect immunofluorescence assay; *ELISA* enzyme immunoassay; *PCR* polymerase chain reaction

**Bold-** significant *p*-values

*p- value <0.05 indicates significant association in the Fisher exact test

^a^Two of the ESH patients had plaque lesions

When compared with the NESH group, the ESH group showed a higher percentage of single lesions (*p* = 0.027) and epithelialized lesions in the first clinical examination (*p* = 0.001). In 30.80% of the patients in the ESH group the lesion was located in the dorsal trunk, and this was observed in 7.60% of the cases in NESH group (*p* = 0.017). There were no significant differences between the median times to wound healing in patients who healed spontaneously with lower limb injuries and those with lesions in other locations, *p* = 0.534.

In the NESH group, there was a predominance of ulcerated lesions (89.60%, *p* = 0.002), the finding of amastigotes in the histopathological exam (62.60%, *p* = 0.005), and positive culture (84.60%, *p* = 0.001). Most of these patients had a single lesion on the lower limbs (42.90%). In the NESH group, the mean time to healing of the lesions in the lower limbs (237 days) was longer than that of lesions at other locations (201 days), *p* = 0.012.

Table 2 reveals the higher positivity in confirmatory parasitological tests [(culture, imprint, PCR and finding of amastigotes in histopathological exams or anti*-Leishmania* immunohistochemistry (IHC)] in the NESH group. Fifty-eight point 8 % of these patients were positive in 3 or more tests, while in the ESH group all cases were positive in only 1 confirmatory exam (*p* = 0.030). Noteworthy is the number of patients in the ESH group positive only in the PCR (*n* = 5; 38.46%).

**Table 2:** Positivity in confirmatory exams^a^ of 445 patients with localized cutaneous leishmaniasis (INI/Fiocruz 2002–2013)

Number of positive tests	Treatment/No treatment				
	NESH		ESH		*p*-value
	n	%	n	%	
1–2	35	41.2	13^b^	100	**0.030**
3–4	50	58.8	0	0	

NESH -Group of treated patients

ESH -Group of patients with early spontaneous healing

**Bold** -significant *p*-value

^a^Imprint; culture; PCR; amastigote in histopathology and/or in anti-*Leishmania* immunohistochemistry

^b^All 13 ESH patients were positive in a single confirmatory test

Considering the concordance concerning the positivity in each test, there were statistically significant differences between the NESH and ESH groups regarding the IFA (*p* = 0.004), ELISA (*p* = 0.002), MST (*p* = 0.05) and culture (*p* = 0.001). In the ESH group we observed differences between the parasitological confirmed cases (non-PCR) and the positivity in the PCR test (*p* = 0.002) (data not shown).

None of the 13 ESH cases presented recurrence of the skin lesions or mucosal involvement during the follow up period. From the 432 patients in the NESH group, 23 relapsed (5.32%) and 11 showed mucosal lesions (2.54%) in follow up.

### Results of survival analysis

**Fig. 2:**
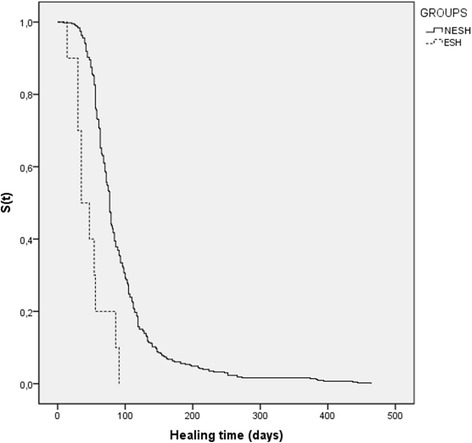
Kaplan-Meier curve; 435 patients with American cutaneous leishmaniasis according to treatment (NESH)/no treatment (ESH) groups and healing time. Continuous line – NESH group: patients with cutaneous leishmaniasis who undergone specific treatment. Dashed line - ESH group: patients with early spontaneous healing. *p*-value <0,001 (INI/Fiocruz, 2002–2013). S(t)- Survival function

The occurrence of clinical cure of the lesion according to the healing time was evaluated through the Kaplan-Meier method (Fig. 2). After the application of censorship criteria, 10 patients were excluded and the analysis were performed on 435 patients (9 patients from ESH group and 426 patients from NESH group).

In all 9 cases of spontaneous healing analyzed by this method, wound healing occurred within approximately 3 months (91 days). As regard to the NESH group, 10 out 426 (2,34%) subjects presented prolonged follow-up (at least 256 days). All 10 cases were censored at the end of the study without presenting the event of interest (healed lesion). There was a statistically significant difference between the groups using the Kaplan-Meier method, evidenced by Peto and Log-rank tests (*p*-values <0.001). Thus, the ESH group showed earlier clinical cure during the observation period.

## Discussion

The diagnosis of ACL in the cases of early spontaneous healing evaluated in this study was confirmed by the identification of the parasite through one of the used parasitological methods. This allowed a study of cases of ACL with spontaneous resolution compared with those who needed specific treatment for the healing of their cutaneous lesions. We found a very low percentage of early spontaneous healing among a historical cohort of patients in a reference center for the disease in Rio de Janeiro, Brazil, where most of the cases are caused by *L. (V.) braziliensis*. Recently, a systematic review of ACL also showed a low percentage (6.0%) of spontaneous healing in CL caused by this parasite [14].

The cases of the ESH group had the highest percentage of single lesions and fewer ulcerated lesions in the initial assessment, and they had less positive results in the confirmatory parasitological tests, differences that were significant in relation to the NESH group. Furthermore, a higher positivity for the presence of parasites by histopathology and culture when the treatment was required was demonstrated, plus a greater percentage of strong reactors in MST. In medical literature, the sensitivity rates of histopathology and isolation in culture medium varies widely [15–17], but the presence of higher parasite loads enhances its detection and thus the parasitological confirmation of the case.

The number of cases of ESH positive only in PCR indirectly suggests that the parasite load tends to be lower in these patients, indicating a balance between the host inflammatory process and the parasite load. A most effective infection control would lead to spontaneous healing. As a result of the probable ability to control the parasite load and the inflammatory process, the cases of ESH had a lower average healing time than that observed in the NESH group. Moreover, the findings suggest that the higher parasite loads in the NESH patients would exert pressure on the inflammatory response, leading to stimulation of the maintenance of the activity of the inflammatory process, which slows its control and subsequently the wound healing. The involvement of the inflammatory response in the formation and maintenance of the leishmaniasis lesions has been demonstrated in a murine model [18]. In humans, although significant difference has not been demonstrated between the amount of the type 1 cytokine interferon gamma (IFNγ) in the peripheral blood of patients with cutaneous leishmaniasis cured after treatment and in the cases with spontaneous healing, in the last ones there was a tendency to higher values [19]. The balance between type 1 and type 2 immunological responses has been suggested as determinant in the evolution of ACL for self-limited or severe forms [20, 21].

The need for specific treatment and the response to the antimonial therapy have been the focus of discussions. Studies in Latin America show significant differences in cure rates with pentavalent antimony (PA) [3, 6, 8, 22–24]. It has also been reported that early treatment of ACL does not prevent the development of ulcers [25, 26] and is associated with higher treatment failure rates [26]. Short evolution times prior to treatment and weak positive MST were both associated with treatment failure [27]. Other factors have also been found to be able to influence the therapeutic response to PA. Among them we can mention the species of the involved parasite, comorbidities, presence of three or more lesions, body weight above sixty-eight kilograms and the irregularity of treatment [5, 28–33]. However, the use of second choice drugs has not shown greater efficacy, or even lower rate of adverse effects. The high toxicity of PA and the increasingly described treatment failure rates has put into discussion the directions for the treatment of ACL.

Criteria for cure, which are essentially clinical, vary according to different authors. This could influence the wide variation in the evaluation of the success of PA therapy. When there is evidence of progression to healing, the extension of the follow up without re-treatment for up to six months is suggested [34, 35]. Therefore we may question the need to propose specific treatment in cases with evidence of progression to early spontaneous healing. Many authors indicate the treatment even in cases of spontaneous regression of the lesions, due to the potential capacity of development of secondary metastatic lesions, especially to mucous membranes, in cases of infection with *L. (V.) braziliensis* [34, 36]. Mucosal leishmaniasis (ML) occurs in a small proportion (3 to 5%) of the ACL cases in Brazil, and can occur several years after the healing of the primary lesion [37]. It is mainly attributed to hematogenous metastases [38]. However, some of these cases share no history of prior cutaneous lesions, and ML is then considered of indeterminate origin [39]. Spontaneous cures and irregular treatments have been considered risk factors for the emergence of ML [10, 39]. However, thorough and longer term follow-up studies are needed. Moreover, in our study, ESH patients did not receive any specific treatment and yearly follow up of 5 post-treatment years did not show any sign of relapse of skin lesions or the appearance of even incipient mucosal lesions in these patients.

Some authors demonstrated that ACL lesions located in the lower limbs needed more time to heal [40, 41]; however, there were greater proportions of healed lesions in this location [16]. Our results also showed that lesions in the lower limbs in treated patients demanded higher healing time than lesions in other locations. Location in the dorsal part of the trunk most rapidly evolved into healing in all patients, and was more often observed in patients with spontaneous resolution. The location in the lower limb did not appear to influence the healing time in patients with early spontaneous healing.

## Conclusions

In conclusion, our results suggest that cases of ACL with progression to early spontaneous healing have an apparently lower parasite load evidenced by the difficulty of parasitological diagnosis during the investigation of the case, since most of them were positive only in the PCR method. Whether this is due to an inoculation of a lower number of parasites during the sandfly bite(s) or a more efficient immune response of the infected persons cannot be defined up to now. Due to obvious signs of healing of the lesions we chose not to initiate specific treatment, since leishmanicidal drugs have great potential for toxicity. Even so, healing of lesions demanded less time than in patients who required treatment. It must be highlighted that even after a long period of follow up post-regression of the lesions, none of the ESH studied cases studied developed recurrence of the lesions or mucosal damage.

The cases of patients with spontaneous resolution in ACL are a challenge in clinical practice. Dermatological/otorhinolaryngological periodic examination of these patients, as well as clear guidance to patients about the signs and symptoms of mucosal lesions are recommended to detect the slight possibility of a relapse.

## Abbreviations

ACL: American cutaneous leishmaniasis
CL: cutaneous leishmaniasis
ELISA: Enzyme Linked Immuno Sorbent Assay
ESH: Early spontaneous healing
FIOCRUZ: Oswaldo Cruz Foundation
HIV: Human Immunodeficiency Virus
IFA: Indirect immunofluorescence assay
IHC: Immunohistochemistry
INI: Evandro Chagas National Institute of Infectious Diseases
LAPCLIN VIGILEISH: Leishmaniasis Surveillance Laboratory
ML: Mucosal leishmaniasis
MST: Montenegro skin test
NESH: non- early spontaneous healing
PA: Pentavalent antimony
PCR: Polymerase Chain Reaction
SPSS: Statistical Package for the Social Sciences
